# Hematopoietic upstream stimulating factor 1 deficiency is associated with increased atherosclerosis susceptibility in LDL receptor knockout mice

**DOI:** 10.1038/s41598-021-95858-y

**Published:** 2021-08-12

**Authors:** Menno Hoekstra, Baoyan Ren, Pirkka-Pekka Laurila, Reeni B. Hildebrand, Jarkko Soronen, Vanessa Frodermann, Zhuang Li, Mariëtte R. Boon, Janine J. Geerling, Patrick C. N. Rensen, Matti Jauhiainen, Miranda Van Eck

**Affiliations:** 1 Gorlaeus Laboratories, Division of BioTherapeutics, Leiden Academic Centre for Drug Research, Leiden University, Einsteinweg 55, 2333 CC Leiden, The Netherlands; 2 Department of Medical Genetics, University of Helsinki, Helsinki, Finland; 3 Genomics and Biobank Unit, National Institute for Health and Welfare, Biomedicum 1, Helsinki, Finland; 4 Pharmaceuticals Division, Bayer Oy BOF-PH-MRA-MA, Medical Affairs PO, Espoo, Finland; 5 Division of Endocrinology, Department of Medicine, Leiden University Medical Center, Leiden, The Netherlands; 6 Einthoven Laboratory for Experimental Vascular Medicine, Leiden University Medical Center, Leiden, The Netherlands; 7 Minerva Foundation Institute for Medical Research, Biomedicum 2U, Helsinki, Finland; 8 Institute for Molecular Medicine Finland, FIMM, Helsinki, Finland

**Keywords:** Biochemistry, Diseases

## Abstract

Total body upstream stimulatory factor 1 (USF1) deficiency in mice is associated with brown adipose tissue activation and a marked protection against the development of obesity and atherosclerotic lesions. Functional expression of USF1 has also been detected in monocytes and monocyte-derived macrophages. In the current study we therefore tested whether selective hematopoietic USF1 deficiency can also beneficially impact the development of atherosclerosis. For this purpose, LDL receptor knockout mice were transplanted with bone marrow from USF1 knockout mice or their wild-type littermate controls and subsequently fed a Western-type diet for 20 weeks to stimulate atherosclerotic lesion development. Strikingly, absence of USF1 function in bone marrow-derived cells was associated with exacerbated blood leukocyte (+ 100%; *P* < 0.01) and peritoneal leukocyte (+ 50%; *P* < 0.05) lipid loading and an increased atherosclerosis susceptibility (+ 31%; *P* < 0.05). These effects could be attributed to aggravated hyperlipidemia, i.e. higher plasma free cholesterol (+ 33%; *P* < 0.001) and cholesteryl esters (+ 39%; *P* < 0.001), and the development of hepatosteatosis. In conclusion, we have shown that hematopoietic USF1 deficiency is associated with an increased atherosclerosis susceptibility in LDL receptor knockout mice. These findings argue against a contribution of macrophage-specific USF1 deficiency to the previously described beneficial effect of total body USF1 deficiency on atherosclerosis susceptibility in mice.

## Introduction

Upstream stimulatory factor 1 (USF1) is a member of basic‐Helix–Loop–Helix–Leucine Zipper family of proteins that modulate the transcriptional activity of their target genes. USF1 was originally identified as a modulator of the adenovirus major late promoter^1–3^. Importantly, USF1 has also emerged as an interesting therapeutic target in the context of metabolic and cardiovascular disease. Several variations in the USF1 gene have been associated with a variety of metabolic traits, i.e. body mass index and plasma glucose and lipid levels, as well as with atherosclerotic cardiovascular disease incidence in humans^4–11^.

In line with the observation that the structure as well as the function of USF1 are well conserved between mice and humans^12^, cardiometabolic associations found in humans can be reproduced in USF1 knockout mice. More specifically, mice lacking USF1 in all endogenous tissues show a significant protection against dyslipidemia, insulin resistance, obesity, hepatic steatosis, and the development of atherosclerotic lesions as compared to wild-type controls^13^. These findings could be translated to humans as the presence of a USF1 allele associated with reduced expression of USF1 was also associated with improved blood lipid levels, insulin sensitivity, and atherosclerosis^13^. The improvement in the metabolic profile in USF1 knockout mice was paralleled by a relatively high brown adipose tissue (BAT) activity and reduced white adipose tissue (WAT) volume^13^. Given that β3-adrenergic receptor-mediated activation of BAT reduces hyperlipidemia and lowers atherosclerosis susceptibility, at least in mice^14^, the positive effect of total body USF1 deficiency on cardiometabolic health, i.e. atherosclerosis susceptibility, was primarily attributed to the associated BAT activation.

However, it should be acknowledged that, apart from its functional expression in brown adipocytes^13^ and liver^15^, USF1 has also been detected in monocytes and monocyte-derived macrophages^16–19^. Macrophages, generated from monocytes infiltrated into the vessel wall in response to endothelial damage, can accumulate LDL after modification by oxidation (oxLDL) or aggregation, and become foam cells, thereby initiating the development of atherosclerotic lesions^20^. Since macrophages as scavenging cells cannot limit the intake of cholesterol-rich lipoprotein particles, they rely fully on active transport of cholesterol out of the cell via ATP-binding cassette transporters such as ABCA1 and ABCG1 to overcome their transformation into foam cells^21^. In vitro studies have recently shown that elimination of USF1 from human THP-1 macrophages induces cellular ABCA1 protein levels and a parallel rise in the rate of cholesterol efflux to wild-type mouse serum^18^. Furthermore USF1 deficient macrophages displayed reduced inflammatory burden with reduced secretion of the pro-inflammatory cytokines monocyte chemoattractant protein-1 and interleukin-1β, thereby supporting the cholesterol efflux process^18^.

Therefore, we hypothesized that the beneficial effect on atherosclerotic cardiovascular disease of total body USF1 deficiency can, at least in part, be attributed to a positive effect on macrophage cholesterol handling. In the current study we tested this hypothesis in atherosclerosis-susceptible mice. More specifically, using bone marrow transplantation we ablated USF1 function in bone marrow-derived cells, including monocytes and macrophages, but not in other metabolic cells such as hepatocytes and (brown) adipocytes in hypercholesterolemic LDL receptor knockout mice and subsequently quantified the extent of atherosclerotic lesion formation after a Western-type diet challenge.

## Results

To determine the effect of selective deletion of USF1 function from bone marrow-derived cells, including macrophages, on atherosclerosis outcome, lethally irradiated atherosclerosis-susceptible LDL receptor knockout mice were transplanted with bone marrow cells from either USF1 knockout mice or their wild-type littermate controls. After recovery from the bone marrow transplantation on a regular chow diet, the two groups of bone marrow recipients were challenged with WTD enriched in cholesterol and fat to stimulate the development of atherosclerotic lesions.

**Figure 1:**
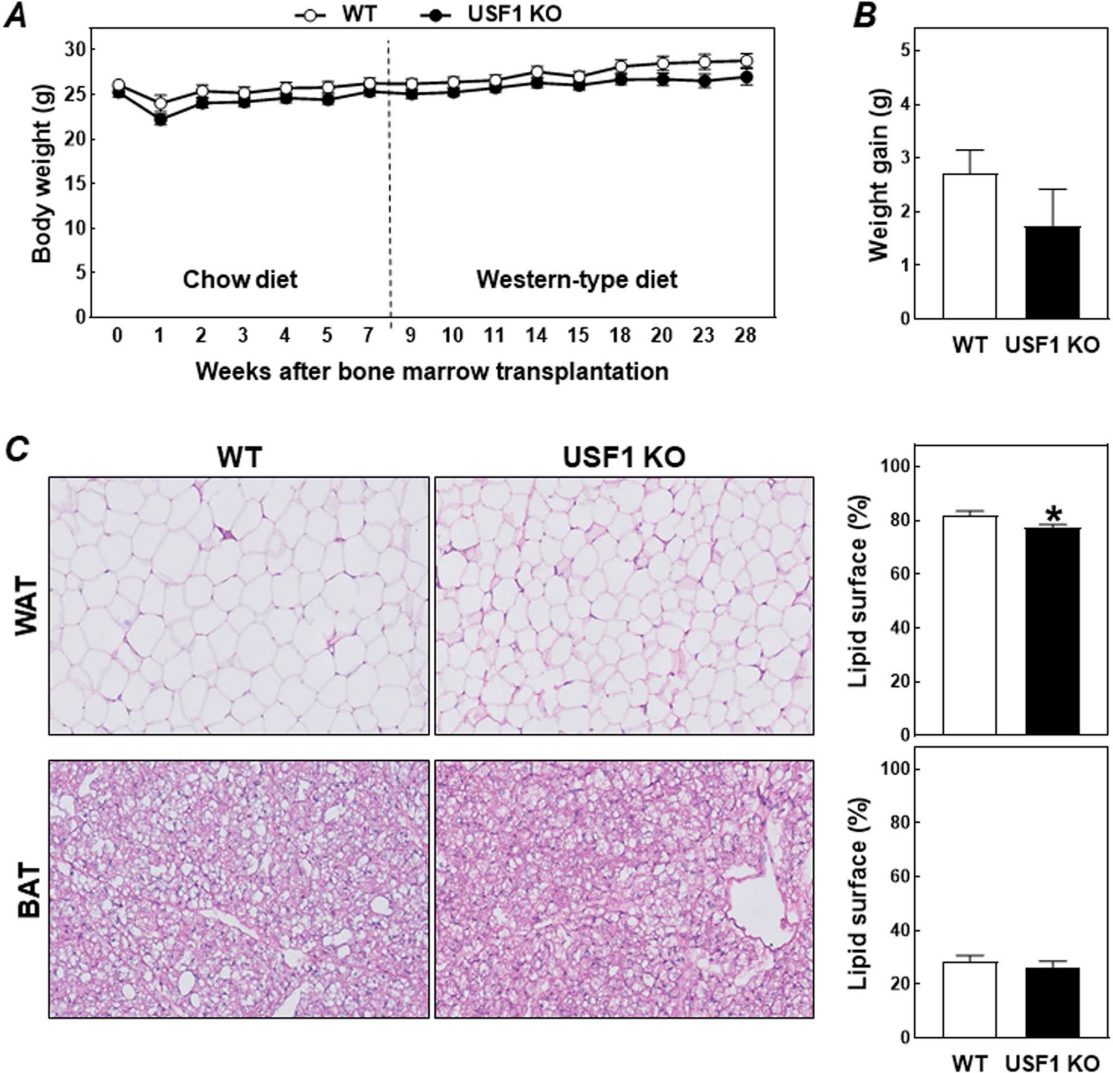
Body weight development (**A**) and total body weight gain (**B**) in LDL receptor knockout mice transplanted with bone marrow from USF1 knockout (USF1 KO) mice or their wild-type (WT) littermate controls. (**C**) Representative images of hematoxylin and eosin-stained sections of BAT and WAT from the two types of bone marrow recipients (left) and quantification of the relative lipid droplet surface areas (right). Data represent the means ± SEM of respectively 11 WT and 16 USF1 KO bone marrow recipients. *P < 0.05 versus WT.

As evident from Fig. 1A, bone marrow-specific USF1 deficiency did not induce a significant difference in body weight development. All LDL receptor knockout recipient mice lost some weight in the first week after the irradiation and bone marrow transplantation procedure. However, from week 2 onwards the two groups of bone marrow recipient mice had reached their original body weight again. Wild-type and USF1 knockout bone marrow recipients ultimately gained on average 2.7 g and 1.7 g (*P* > 0.05), respectively, during the complete course of the study, i.e. after 8 weeks regular chow diet and 20 weeks of Western-type diet feeding (Fig. 1B). A minor decrease in gonadal WAT lipid content was noted (− 5%, *P* < 0.05; Fig. 1C), while the relative surface area of BAT sections covered by lipid droplets was not significantly different in USF1 bone marrow recipients as compared to wild-type bone marrow recipients (Fig. 1C). In further support of the notion that bone marrow-specific USF1 deficiency generally does not influence BAT functionality, relative mRNA expression levels of the thermogenic protein uncoupling protein 1 (UCP1) were also not different between the two groups of bone marrow transplanted LDL receptor knockout mice (relative levels as compared to housekeeping mRNA expression: 12.7 ± 1.4 for USF1 knockout recipients (N = 10) versus 11.1 ± 1.0 for WT knockout recipients (N = 8), respectively; *P* > 0.05).

**Figure 2:**
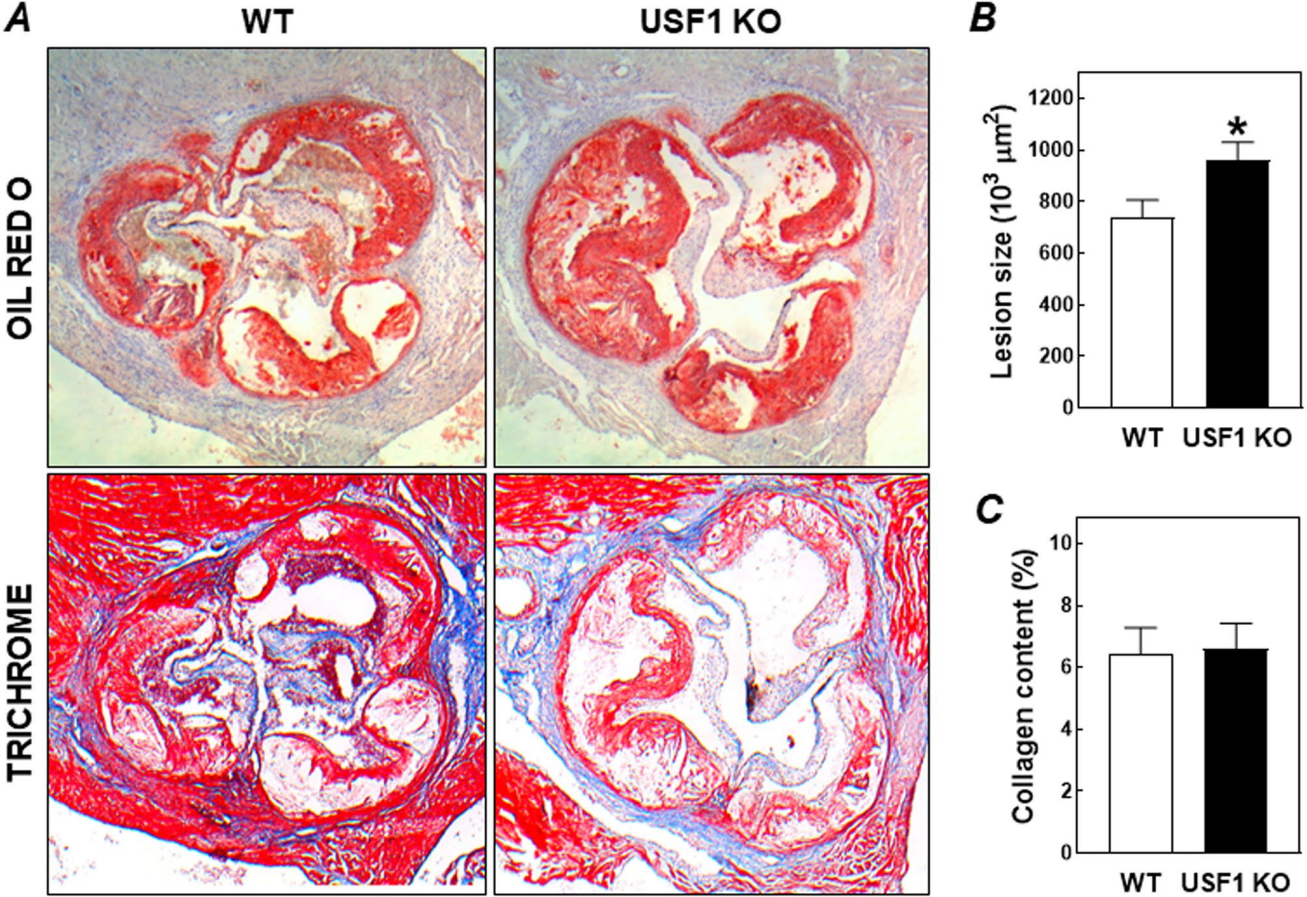
(**A**) Representative images of Oil red O- and Masson's Trichrome-stained aortic root sections from LDL receptor knockout mice transplanted with bone marrow from USF1 knockout (USF1 KO) mice or their wild-type (WT) littermate controls. Quantifications of the aortic root total plaque area (**B**) and lesional collagen contents (**C**). Data represent the means ± SEM of respectively 11 WT and 16 USF1 KO bone marrow recipients. *P < 0.05 versus WT.

Hearts of the mice were collected for cryosectioning and quantification of the extent of atherosclerotic lesion development in the aortic root. Oil red O-staining revealed extensive lesion formation within the aortic root in both groups of bone marrow recipient mice (Fig. 2A). In contrast to our hypothesis that macrophage USF1 deficiency would attenuate the progress of atherosclerosis, the outcome of ablation of USF1 function in bone marrow cells was actually opposite and associated with a moderately increased atherosclerosis susceptibility. More specifically, the average lesion size in USF1 knockout bone marrow recipients was 962 ± 67 × 10^3^ µm^2^ versus 737 ± 69 × 10^3^ µm^2^ in wild-type bone marrow controls (*P* < 0.05; Fig. 2B). Trichrome staining showed that the plaques in both groups of mice were relatively collagen poor, without significant differences between the two groups (6–7% of total plaque area; Fig. 2C). In accordance with a very advanced stage of lesion development, the Trichrome staining also verified that atherosclerotic plaques in both groups of bone marrow recipients generally contained little macrophage foam cells (pink-stained cells) and were rather rich in cell-deprived/necrotic areas surrounded by thick layers of smooth muscle cells (red staining).

**Figure 3:**
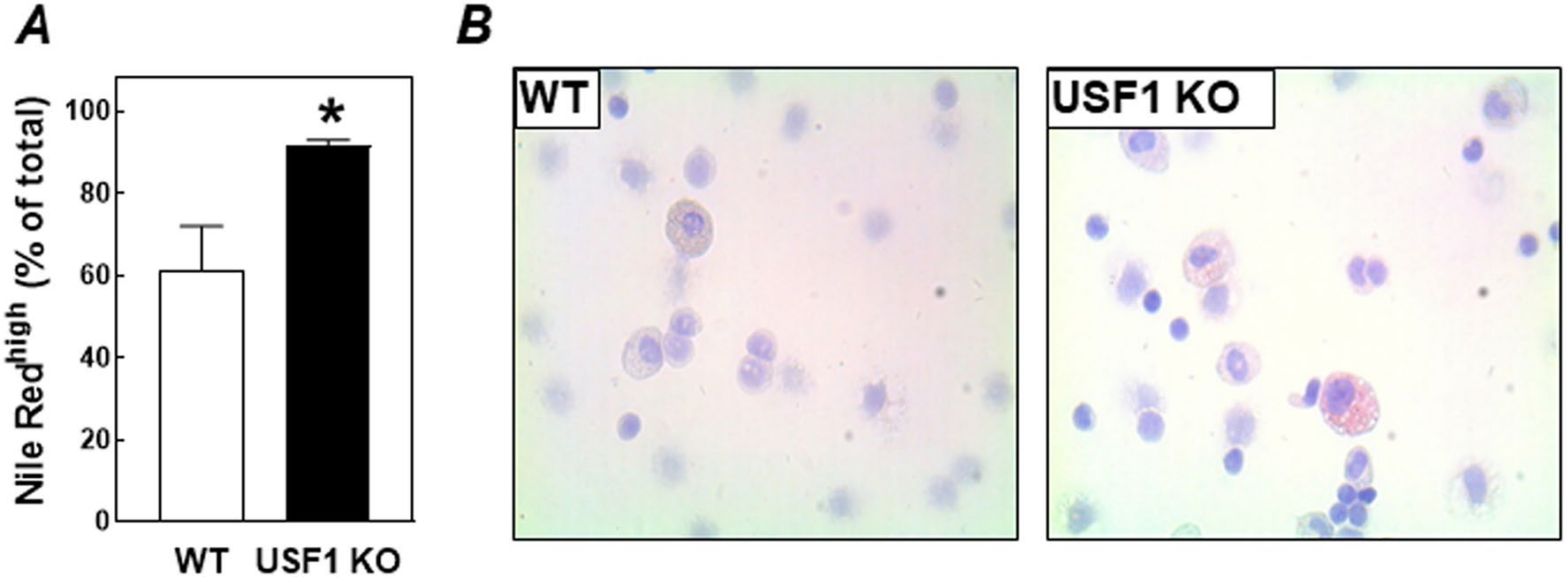
(**A**) The percentage of peritoneal leukocytes isolated from LDL receptor knockout mice transplanted with bone marrow from USF1 knockout (USF1 KO) mice or their wild-type (WT) littermate controls that stained positive using Nile Red for the presence of neutral lipid accumulation. Data represent the means ± SEM of 6 randomly chosen WT and USF1 KO bone marrow recipients. *P < 0.05 versus WT. (**B**) Representative images of Oil red O-stained cytospins of isolated peritoneal leukocytes.

Our previous bone marrow transplantation studies in LDL receptor knockout mice have suggested that, under WTD feeding conditions, the extent of macrophage lipid accumulation in peritoneal leukocyte fractions acts as a sensitive predictor for atherosclerosis susceptibility^22–24^. In accordance, flow cytometric analysis of isolated peritoneal leukocytes showed that the fraction of cells that stained highly positive for the fluorescent neutral lipid dye Nile Red was significantly larger (Nile Red^high^; + 50%; *P* < 0.05) in USF1 knockout bone marrow-transplanted mice as compared to wild-type bone marrow transplanted mice (Fig. 3A). In further support, Oil red O-positive lipid-rich foam cells were almost only visible in the cytospins from peritoneal leukocyte fractions of USF1 knockout bone marrow recipients (Fig. 3B).

**Figure 4:**
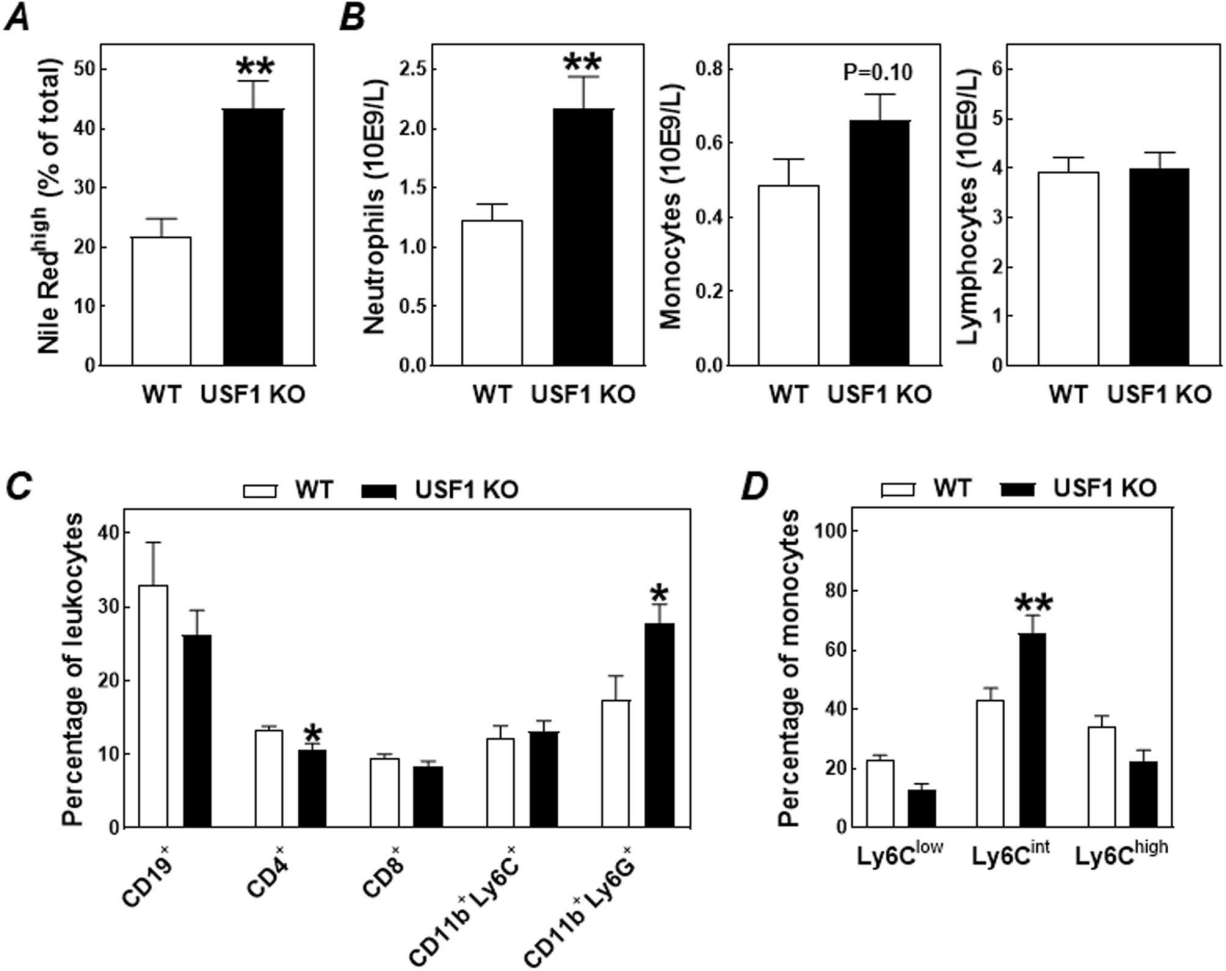
The percentage of blood leukocytes that stained positive using Nile Red for the presence of neutral lipid accumulation (**A**), total blood counts of neutrophils, monocytes, and lymphocytes (**B**), and the distribution of blood monocytes over the different Ly6C-driven activation states (**C**) in LDL receptor knockout mice transplanted with bone marrow from USF1 knockout (USF1 KO) mice or their wild-type (WT) littermate controls. Data in panels (**A**, **C**) represent the means ± SEM of 6 randomly chosen WT and USF1 KO bone marrow recipients, whilst the data in panel (**B**) represent the means ± SEM of respectively 11 WT and 16 USF1 KO bone marrow recipients. **P < 0.01 versus WT.

Blood collected at sacrifice was analyzed to verify whether the relatively high neutral lipid levels in USF1 knockout macrophages were also already present at the (blood) monocyte differentiation stage. In agreement, a marked increase was detected through flow cytometry in the percentage of Nile Red^high^ leukocytes in the blood of USF1 knockout bone marrow recipients as compared to wild-type bone marrow recipients (+ 100%; *P* < 0.01) (Fig. 4A). Parallel routine hematological analysis showed no change in absolute blood lymphocyte counts in LDL receptor knockout mice transplanted with USF1 knockout bone marrow (Fig. 4B). The increase in leukocyte lipid levels, however, did coincide with a significant rise in the blood neutrophil count (+ 64%; *P* < 0.01; Fig. 4B) and a trend towards an increase in circulating monocyte numbers (+ 35%; *P* = 0.10; Fig. 4B). Flow cytometric analysis further verified that the marked increase in absolute neutrophil counts in USF1 knockout bone marrow recipients translated into a shift in the relative blood leukocyte distribution pattern. The blood leukocyte population from USF1 knockout bone marrow recipients contained relatively lower numbers of CD4^+^ helper and CD8^+^ cytotoxic T cells and CD19^+^ B cells in the context of a significantly higher CD11b^+^Ly6G^+^ neutrophil fraction (Fig. 4C). Relative CD11b^+^Ly6C^+^ monocyte counts were not different between the two groups of mice (Fig. 4C). However, bone marrow USF1 deficiency was associated with a monocyte phenotype shift towards the pro-inflammatory monocyte fraction expressing the cell surface marker Ly6C at an intermediate level (Ly6C^int^; + 52%; P < 0.01; Fig. 4D).

**Figure 5:**
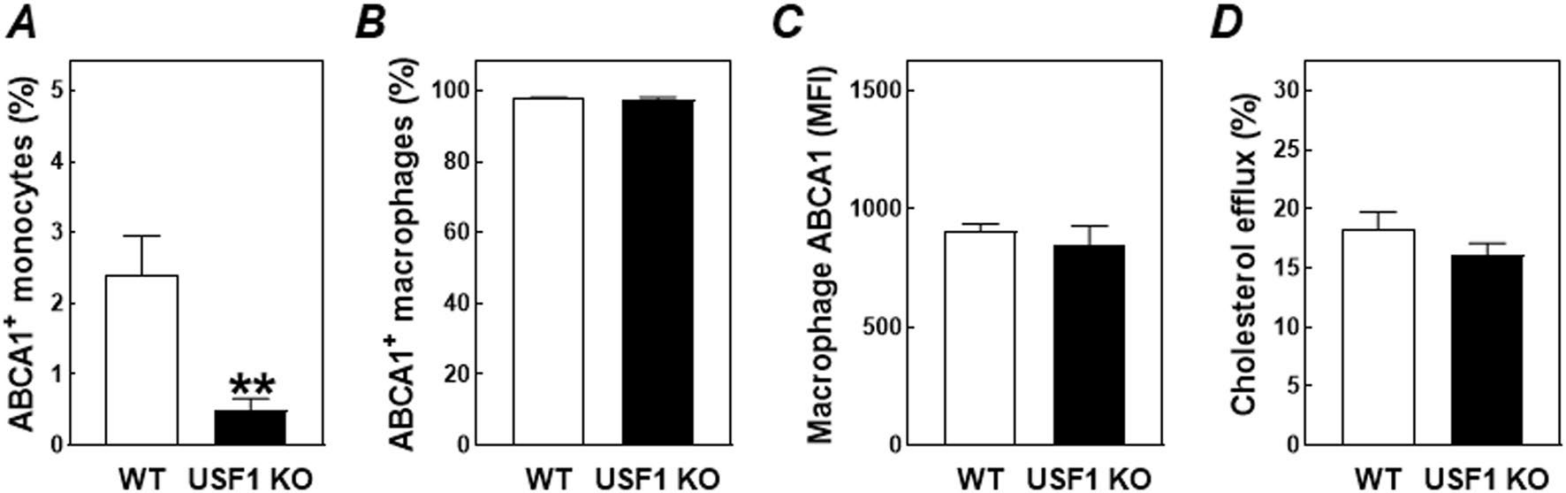
The percentage of blood monocytes (**A**) and peritoneal macrophages (**B**) that stained positive for the presence of ABCA1 protein, and (**C**) relative surface protein expression levels of ABCA1 on peritoneal macrophages in LDL receptor knockout mice transplanted with bone marrow from USF1 knockout (USF1 KO) mice or their wild-type (WT) littermate controls. (**D**) The extent of cholesterol efflux from WT and USF1 KO bone marrow-derived macrophages to the exogenous acceptor apolipoprotein A1. Data in panels (**A**–**C**) represent the means ± SEM of 6 randomly chosen WT and USF1 KO bone marrow recipients, whilst the in vitro data in panel (**D**) represent the means ± SEM of 4 cell culture wells. **P < 0.01 versus WT.

Since data from Ruuth et al.^18^ suggested a possible effect of USF1 on macrophage ABCA1 activity, we quantified ABCA1 protein levels on the blood monocytes and peritoneal macrophages. As can be appreciated from the flow cytometry results in Fig. 5A, the fraction of blood monocytes expressing ABCA1 was already very low in mice transplanted with wild-type bone marrow (2.4 ± 0.6%) and decreased even further in those that had received USF1 knockout bone marrow (0.5 ± 0.2%; *P* < 0.01). In contrast, ABCA1 expression was similarly high in F4/80^+^ peritoneal macrophages, both in terms of the relative number of cells expressing the transporter (97–98% positive of total; Fig. 5B) and the absolute amount of protein present on the individual macrophages (similar ABCA1 mean fluorescent intensity; Fig. 5C). The rate of [^3^H]cholesterol efflux from bone marrow-derived macrophages towards the exogenous acceptor apolipoprotein A1 (apoA1) in vitro was also not affected by a change in the USF1 genotype (Fig. 5D). From these combined findings it can be suggested that the bone marrow USF1 deficiency-associated susceptibility to macrophage foam cell formation and the development of atherosclerotic lesions is likely not due to a change in the ability of USF1 knockout macrophages to remove excess cellular cholesterol through ABCA1-mediated efflux.

**Figure 6:**
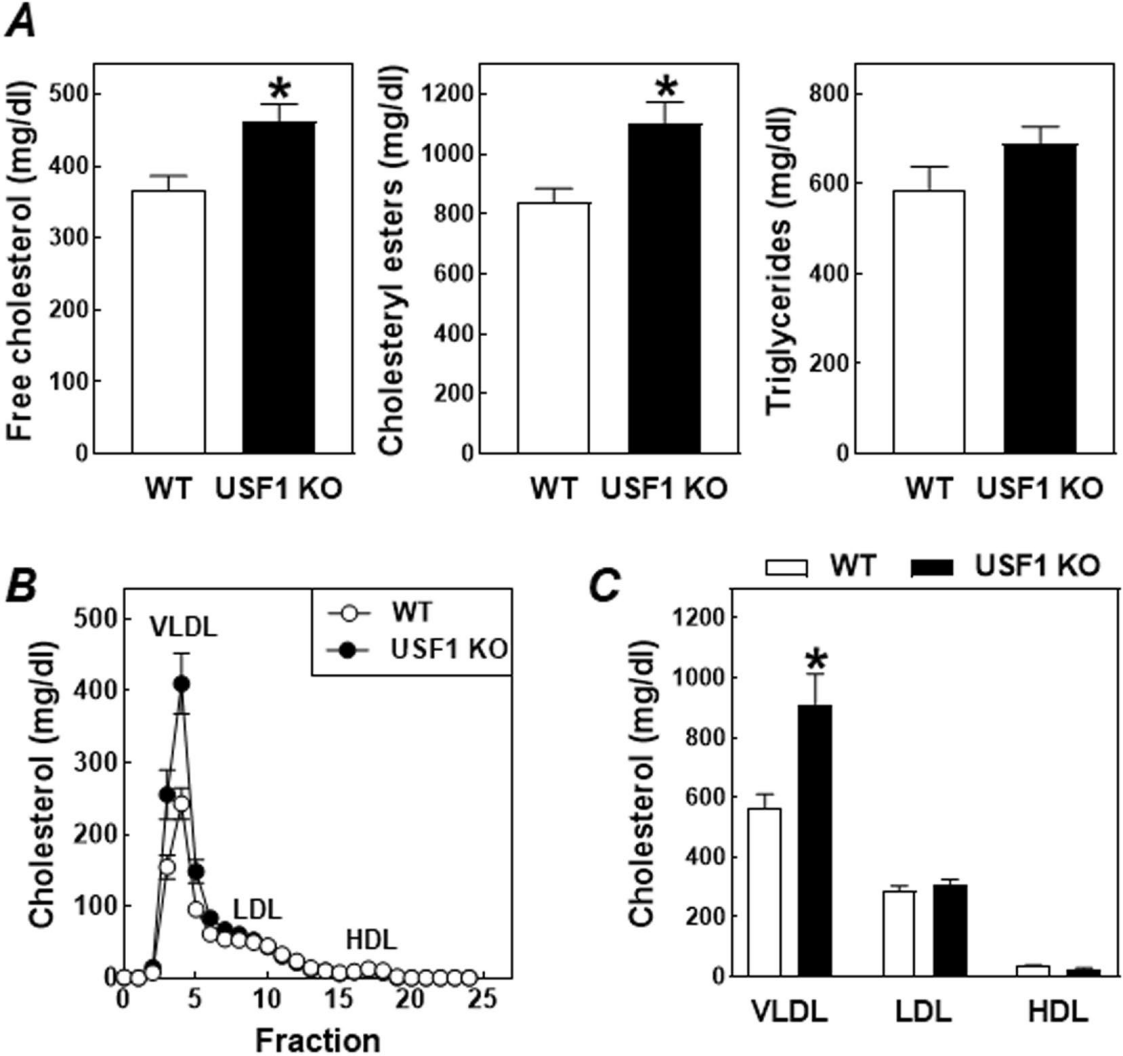
Plasma levels of free cholesterol, cholesteryl esters and triglycerides (**A**) and the distribution of total cholesterol over the different lipoproteins (**B**,**C**) in LDL receptor knockout mice transplanted with bone marrow from USF1 knockout (USF1 KO) mice or their wild-type (WT) littermate controls. Fractions 1–6 represent VLDL, fractions 7–13 LDL, and fractions 14–22 HDL. Data represent the means ± SEM of respectively 11 WT and 16 USF1 KO bone marrow recipients. *P < 0.05 versus WT.

The levels of cholesterol associated with plasma very-low-density lipoproteins (VLDL) and their remnants are a primary determinant for atherosclerosis susceptibility in LDL receptor knockout mice^25^. In support of the suggestion that the higher foam cell susceptibility could be related to exacerbated hypercholesterolemia, plasma free cholesterol and cholesteryl ester levels were respectively 33% (*P* < 0.001) and 39% (*P* < 0.001) higher in USF1 knockout bone marrow recipients as compared to wild-type bone marrow recipients after 20 weeks of Western-type diet feeding (Figs. 6A). Plasma triglyceride levels in USF1 knockout bone marrow recipients (689 ± 38 mg/dl) were, however, not significantly increased as compared to wild-type bone marrow recipients (588 ± 50 mg/dl; Fig. 6A). Fast performance liquid chromatography-based fractionation of the different lipoproteins in whole plasma specimens validated that the increase in total plasma cholesterol levels could be attributed to a specific increase in total cholesterol associated with VLDL particles (Fig. 6B, C).

**Figure 7:**
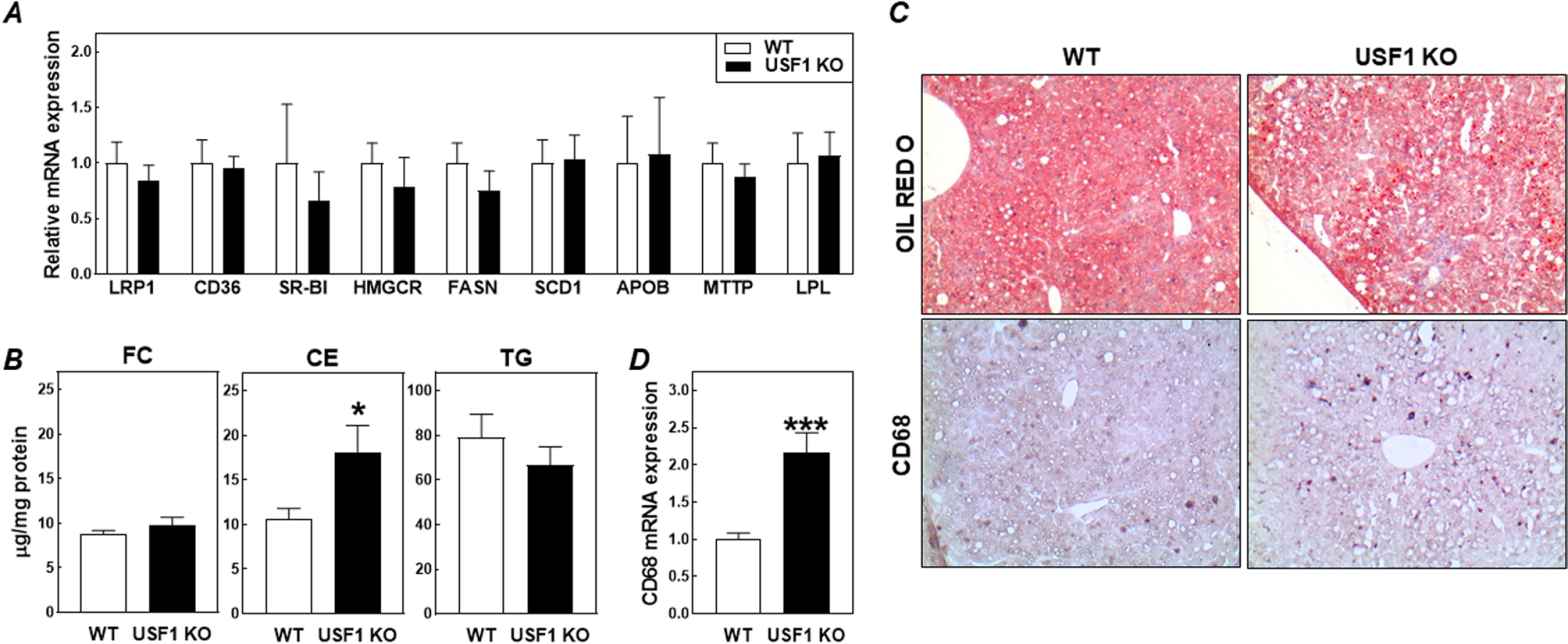
Hepatic relative mRNA expression levels of genes involved in liver lipid metabolism (**A**), liver free cholesterol (FC), cholesteryl ester (CE), and triglyceride (TG) contents (**B**), and hepatic CD68 mRNA expression levels (**C**) in LDL receptor knockout mice transplanted with bone marrow from USF1 knockout (USF1 KO) mice or their wild-type (WT) littermate controls. (**D**) Representative images of liver sections from the two types of bone marrow recipients that were either stained for neutral lipids using Oil red O or for the presence of macrophages using an antibody directed against CD68. Data in panels (**A**, **C**) represent the means ± SEM of 7 randomly chosen WT and USF1 KO bone marrow recipients, whilst the data in panel (**B**) represent the means ± SEM of 8 randomly chosen WT and USF1 KO bone marrow recipients. *P < 0.05, ***P < 0.001 versus WT.

The liver is a major contributor to steady state VLDL-cholesterol levels in the circulation of LDL receptor knockout mice as it facilitates the de novo synthesis of triglyceride-rich VLDL particles, contributes to lipoprotein lipase (LPL)-mediated VLDL-triglyceride lipolysis, and is able to clear cholesterol-rich VLDL remnants from the circulation. Hepatic mRNA expression levels of the LDL receptor-related protein 1 (LRP1) and class B scavenger receptors CD36 and SR-BI were not significantly different between the two groups of transplanted LDL receptor knockout mice (Fig. 7A), suggesting a similar uptake of cholesterol from lipoproteins by the liver. The de novo synthesis of cholesterol and fatty acids and subsequent packaging of these lipid species in VLDL particles was likely also not changed as judged from the unchanged mRNA expression levels of apolipoprotein B (APOB) and microsomal triglyceride transfer protein (MTTP) and the sterol regulatory element binding protein (SREBP) target genes HMG-CoA reductase (HMGCR), fatty acid synthase (FASN), and stearoyl-CoA dismutase 1 (SCD1) (Fig. 7A). Furthermore, the bone marrow USF1 genotype did not influence hepatic LPL mRNA expression levels (Fig. 7A). Free cholesterol and triglyceride contents of the livers were also not different between the two groups of mice (Fig. 7B). However, as evident from Fig. 7B, the increase in plasma VLDL-cholesterol levels in USF1 knockout bone marrow recipients did coincide with a marked increase in hepatic cholesteryl ester stores (+ 72%; *P* < 0.05). Oil red O staining on liver cryosections validated the excessive presence of neutral lipid deposits in mice reconstituted with USF1 knockout bone marrow (Fig. 7C). Notably, the mRNA expression level of the macrophage gene marker CD68 was > twofold higher (*P* < 0.001) in livers of USF1 knockout bone marrow recipients (Fig. 7D). As can be appreciated from the representative images in Fig. 7C, immunohistochemical staining verified a rise in the number of CD68-positive tissue macrophages, i.e. Kupffer cells, in liver cryosections of USF1 knockout bone marrow recipients. The bone marrow-specific USF1 deficiency-associated hypercholesterolemia was thus paralleled by the development of steatohepatitis, i.e. accumulation of cholesteryl esters and inflammatory macrophages within the liver, which could not be explained by changes in hepatic transcript levels of relevant cholesterol metabolism-related proteins. As steatohepatitis has been shown to be an independent risk factor for the development of atherosclerotic cardiovascular disease^26^, the increased steatohepatitis could contribute to the moderate increase in atherosclerotic lesions observed upon USF1 deficiency in our specific model.

## Discussion

In this study it was shown that selective ablation of USF1 in bone marrow-derived cells, including macrophages, is associated with augmented hypercholesterolemia and foam cell development and a parallel increase in the atherosclerosis susceptibility in LDL receptor knockout mice. These findings (1) argue against a direct contribution of macrophages to the previously described beneficial effect of total body USF1 deficiency on atherosclerosis susceptibility^13^ and rather (2) suggest that when targeting USF1 in the context of atherosclerotic cardiovascular disease one should actually try to maintain optimal USF1 levels in macrophages to overcome exacerbation of the hypercholesterolemia and macrophage foam cell extent. Although macrophages could contribute to development of atherosclerosis in the whole-body knockout, this might require the presence of a metabolically favorable milieu, featuring reduced adiposity, insulin resistance, systemic inflammation, and hepatic steatosis observed in the whole-body knockouts^13^, but not in the bone-marrow specific USF1 knockouts.

An interesting finding of our studies is that the increase in foam cell extent in response to macrophage USF1 deficiency was not caused by a diminished ABCA1 expression or cholesterol efflux activity. This observation does not concur with the previous observation of Ruuth et al. that USF1 acts as a negative regulator of ABCA1 expression in human THP-1 macrophages. In humans, USF1 affects ABCA1 transcription through an interaction with the E-box motif in the ABCA1 proximal promoter region^27^. A check of the nucleotide sequence overlap between the human and mouse promoters showed that the region where the E-box motif is located is not highly conserved (only ~ 50% overlap)^28^. It is therefore possible that USF1 is not able to execute its negative impact on ABCA1 transcription in mice due to the lack of the required regulatory site, explaining the absence of an effect in our current in vivo experimental setting. However, dedicated follow-up studies using reporter constructs containing the human and mouse ABCA1 promoter regions are warranted to validate this possibility.

A change in macrophage ABCA1 expression apparently did not underlie the enhanced susceptibility for the development of macrophage foam cells and atherosclerotic lesions in this model of USF1 deficient bone-marrow transplantation. We therefore anticipate that the increase in plasma cholesterol levels represents the driving force behind the aggravated atherosclerosis susceptibility found in USF1 knockout bone marrow recipients. The higher plasma cholesterol levels coincided with excessive storage of cholesteryl esters and macrophage infiltration in the liver, suggesting that a change in hepatic cholesterol homeostasis may underlie the development of the exacerbated hypercholesterolemia. Importantly, the cholesterol fraction in circulating VLDL particles was significantly elevated, likely as a result of further lipolysis of remnant particles with a prolonged circulation time. These cholesterol-enriched remnant particles are known to be atherogenic as they stimulate the development of foam cells^29^. However, based upon the studies by Wouters et al., it can also be suggested that the relatively higher hepatic inflammation state—i.e. increased liver macrophage content—in USF1 knockout bone marrow recipients may be secondary to the higher plasma VLDL-cholesterol levels rather than to the higher degree of hepatic cholesteryl ester accumulation^30^. Notably, clinical studies have suggested that an increase in hepatic steatosis extent generally coincides with a higher systemic inflammation status^31–34^. As such, the aggravated liver steatosis phenotype can theoretically explain why bone marrow USF1 deficiency was associated with a rise in the blood concentration of pro-inflammatory monocytes and neutrophils. Studies by Khoury et al. have suggested that, amongst fatty liver disease patients, a higher neutrophil-to-lymphocyte ratio can serve as a clinical predictor of a more advanced hepatic steatosis phenotype, i.e. non-alcoholic hepatosteatitis^35^. In support, the higher blood neutrophil number was paralleled by a marked increase in the number of tissue macrophages in livers of mice reconstituted with USF1 bone marrow as compared to those that were transplanted with wild-type bone marrow.

Despite the fact that the increased hepatic cholesteryl ester load can possibly underlie the exacerbated hypercholesterolemia and associated atherosclerosis susceptibility, the question remains why an exclusive lack of USF1 in bone marrow-derived cells is associated with a higher steatosis degree as we did not detect a change in the hepatic expression of genes involved in cholesterol synthesis and lipoprotein uptake. Given that hepatocytes are not replaced after bone marrow transplantation, a direct effect of hematopoietic USF1 deficiency on hepatocyte functioning can be excluded. Previous studies have shown that a change in the activation status of Kupffer cells may affect liver lipid metabolism and hepatic steatosis extent^36–38^. It can therefore be suggested that a change in bone marrow USF1 genotype translated into an altered (tissue) macrophage functioning locally in the liver. The studies by Zheng et al. have indicated that specifically the conversion of tissue macrophages, i.e. Kupffer cells, into the M2 (anti-inflammatory/resolving) type contributes to the protective effect within the context of hepatic steatosis^38^. Gene expression levels of the M1 macrophage markers TNF-alpha and interleukin-6 were, unfortunately, too low to be reliably detected in all liver samples of our two groups of bone marrow transplanted mice (Ct ≥ 35). We are thus unable, using the biological material obtained at sacrifice, to—in hindsight—properly judge the phenotype of the wild-type and USF1 knockout Kupffer cells in our experimental setting. The observation by Egan et al. that USF1 is able to bind the mannose receptor promoter to modulate its transcriptional activity in rats and humans but not in mice^39^ suggests that the impact of USF1 deficiency on macrophage polarization may vary significantly between species. As a result, in contrast to the whole-body USF1 knockout mice in which most of the effects translated to humans, one must conclude that it may be more difficult to translate findings on the regulatory role of USF1 on inflammation and metabolism from bone-marrow specific mouse models to the human context.

In conclusion, our bone marrow transplantation study has shown that macrophage USF1 deficiency is associated with the presence of steatohepatitis, aggravated hyperlipidemia, and increased atherosclerosis susceptibility in LDL receptor knockout mice. Our findings highlight that the anti-atherogenic effect of total body USF1 deficiency in mice is not mimicked by bone marrow-specific USF1 gene ablation, probably due to the different impact on (brown) adipose tissue functioning. However, since it appears that the impact of USF1 deficiency on macrophage cholesterol efflux may be different across species, we still regard it possible that macrophages do contribute significantly to the overall beneficial effect of total body USF1 deficiency on metabolic and atherosclerotic cardiovascular disease risk in humans.

## Methods

### Animals and bone marrow transplantation

LDL receptor knockout mice on a C57BL/6J background were purchased from the Jackson Laboratories and bred under standard laboratory conditions at the Gorlaeus Laboratories in Leiden, the Netherlands. USF1 knockout mice^13^ and their wild-type littermates (both C57BL/6J background) were bred at the National Institute for Health and Welfare and University of Helsinki. All animal work was approved by the Dutch Ethics Committee and regulatory authority at Leiden University and was carried out in compliance with the Finnish and Dutch government guidelines, the Directive 2010/63/EU of the European Parliament on the protection of animals used for scientific purposes, and the ARRIVE guidelines.

Bones were harvested from female USF1 knockout mice and wild-type littermates in Helsinki and transported to Leiden in Dulbecco's modified Eagle medium (DMEM). Within 36 h after collection of the bones, bone marrow was isolated. One day after lethal irradiation (Röntgen, 8 Gy), male LDL receptor knockout recipients (~ 12 weeks old) were transplanted with the different bone marrow genotypes through intravenous tail vein injection of 5 × 10^6^ cells (N = 20 per bone marrow genotype). Bone marrow recipients were allowed to recover for 8 weeks on a chow diet (RM3; Special Diet Services). Subsequently, all mice were fed a Western-type diet (WTD) containing 0.25% cholesterol, 15% cocoa butter and 1% corn oil (SDS, Sussex, UK) to stimulate the development of atherosclerotic lesions. During the study, several mice had to be killed due to the development of severe fighting wounds and/or diarrhea. As such, after 20 weeks of WTD feeding only 11 wild-type bone marrow and 16 USF1 knockout bone marrow recipient mice were still alive, respectively. These remaining mice were anaesthetized using a mix of xylazine, ketamine and atropine. Blood was collected by tail cut for lipid analysis or by retro-orbital bleeding for flow cytometric analysis and routine hematological testing. Subsequently, the animals were perfused with PBS, and the heart and other organs were collected for further research.

### Atherosclerotic lesion analysis in aortic root

Hearts of the transplanted LDL receptor knockout recipients (obtained at sacrifice, i.e. after the 20 week WTD challenge) were fixed in 4% Shandon Zinc Formal-Fixx (Thermo Fisher Scientific, 9990245) for 24 h and subsequently embedded in Tissue-Tek O.C.T. compound (Sakura Finetek, USA) until further processing. Cryosections (7 μm) at the level of the aortic sinus were obtained using a Leica CM3050s cryostat. Atherosclerotic plaques were stained with Oil Red O for neutral lipids and Masson’s Trichrome to identify collagen. Plaque area (in µm^2^) and lesional collagen content quantifications were performed blinded using a Leica image analysis system (Leica Ltd, Cambridge, UK).

### Plasma lipid determination

After 4 h of fasting, blood was collected via tail sampling in potassium-EDTA microvette CB 300 tubes (Sarstedt, Nümbrecht, Germany) and centrifuged at 2000 rpm for 5 min to separate out the plasma. Free cholesterol and total cholesterol levels were determined in plasma using colorimetric assays as previously described^40^. Furthermore, plasma lipoprotein profile analysis was performed by fast protein liquid chromatography (FPLC) using a high-resolution size-exclusion chromatography Superose 6 HR column (3.2 × 30 mm; Smart-System, Pharmacia, Uppsala, Sweden).

### Macrophage cholesterol efflux assay

Bone marrow-derived macrophages, generated through exposure of bone marrow cells to M-CSF-containing L929 cell supernatant for 7 days, were allowed to adhere overnight to 24-well cell culture plates in DMEM containing 10% fetal calf serum. Subsequently, the macrophages were exposed to 0.5 µCi [^3^H]cholesterol (Perkin Elmer, Waltham, MA, US) in DMEM with 0.2% fatty acid free BSA for 24 h. Finally, the ability of the macrophages to efflux the incorporated radioactive cholesterol towards DMEM with 0.2% fatty acid free BSA containing the cholesterol acceptor, essentially lipid-free human apolipoprotein A1 (10 μg/mL; Calbiochem) was measured after 24 h incubation and calculated as the percentage of radioactivity in the medium compared with the total amount of radioactivity in the medium and cells.

### Hepatic lipid extraction

Total lipids were extracted from liver samples of the transplanted LDL receptor knockout recipients (obtained at sacrifice, i.e. after the 20 week WTD challenge) using the Bligh and Dyer method^41^ and dissolved in 2% Triton X-100. The free cholesterol, cholesteryl ester and triglyceride content in the homogenate were measured using the colorimetric assays and divided by the protein content as determined using a BCA assay^42^, and expressed as “µg lipid/mg protein”.

### Adipose tissue lipid surface area quantification

Paraffin embedded sections (5 µm) from gonadal WAT and interscapular BAT of the transplanted LDL receptor knockout recipients (obtained at sacrifice, i.e. after the 20 week WTD challenge) were prepared and stained by hematoxylin and eosin. The lipid droplet-positive area as percentage of the total WAT and BAT area was quantified using Image J software (version 1.47)^14^.

### Hematological analysis and flow cytometry

Blood leukocyte counts of the transplanted LDL receptor knockout recipients (obtained at sacrifice, i.e. after the 20 week WTD challenge) were analysed using an automated Sysmex XT-2000iV Veterinary Haematology analyser (Sysmex Corporation). Fluorescent activated cell sorting analysis was performed on a FACS Canto II flow cytometer (BD Biosciences, Mountain View, CA) to detect cell surface markers on leukocytes. For this purpose, blood samples and peritoneal cells were treated with ammonium–chloride–potassium erythrocyte lysing buffer for 2 × 5 min and, after washing the pellet with PBS supplemented with 1% FBS, leukocytes were stained with a mix of antibodies directed against CD11b (FITC-conjugated), Ly6C (PE-conjugated), and Ly6G (APC-conjugated) to identify CD11b^+^/Ly6G^+^ neutrophils and CD11b^+^/Ly6C^+^ monocytes or an antibody cocktail specifically targeting CD19 (FITC-conjugated), CD4 (PE-conjugated) or CD8 (PerCP-conjugated) to quantify B cell, helper T cell and cytotoxic T cell numbers, respectively. After an additional wash, the stained cells were loaded into the flow cytometer. All antibodies used were from eBioscience (Thermo Fisher Scientific). Nile red (Sigma-Aldrich, USA) was used to detect lipid-rich leukocytes. Data were analysed using FlowJo Software v10 (TreeStar Inc). See Supplemental Fig. 1 for the gating strategy/representative gating plots. The mean fluorescent intensity (MFI) was used as measure for cellular relative protein expression levels.

### Gene expression analysis by real time PCR

Total RNA was isolated from randomly chosen subgroups of liver and BAT samples of the transplanted LDL receptor knockout recipients obtained at sacrifice, i.e. after the 20 week WTD challenge. cDNA was synthesized using RevertAid M-MuLV reverse transcriptase according to the manufacturer’s protocol (Thermo Scientific, USA). Relative mRNA expression levels were measured on a 7500 Fast Real-Time PCR system (Applied Biosystems) using SensiMix SYBR green technology. The average expression of the housekeeping genes GAPDH, HPRT and 36B4 was used as a reference for calculation of the relative expression levels of genes of interest. Primer sequences are available upon request.

### Statistical analysis

All values are expressed as means ± SEM. Differences between the groups were statistically analysed with the unpaired Student’s t-test or two-way ANOVA with Bonferroni post-test using GraphPad Prism software (San Diego, CA, USA). Welch correction was applied in case of unequal variances in the dataset. *P* values < 0.05 were considered statistically significant.

## Supplementary Information

Supplementary Figure 1.

## Data Availability

The datasets generated during and/or analysed during the current study are available from the corresponding author on reasonable request.
