# Supplementary figures and images for: Hematopoietic upstream stimulating factor 1 deficiency is associated with increased atherosclerosis susceptibility in LDL receptor knockout mice
Source: Sci Rep. 2021 Aug 12;11:16419. doi: 10.1038/s41598-021-95858-y (PMC8361089; doi:10.1038/s41598-021-95858-y)

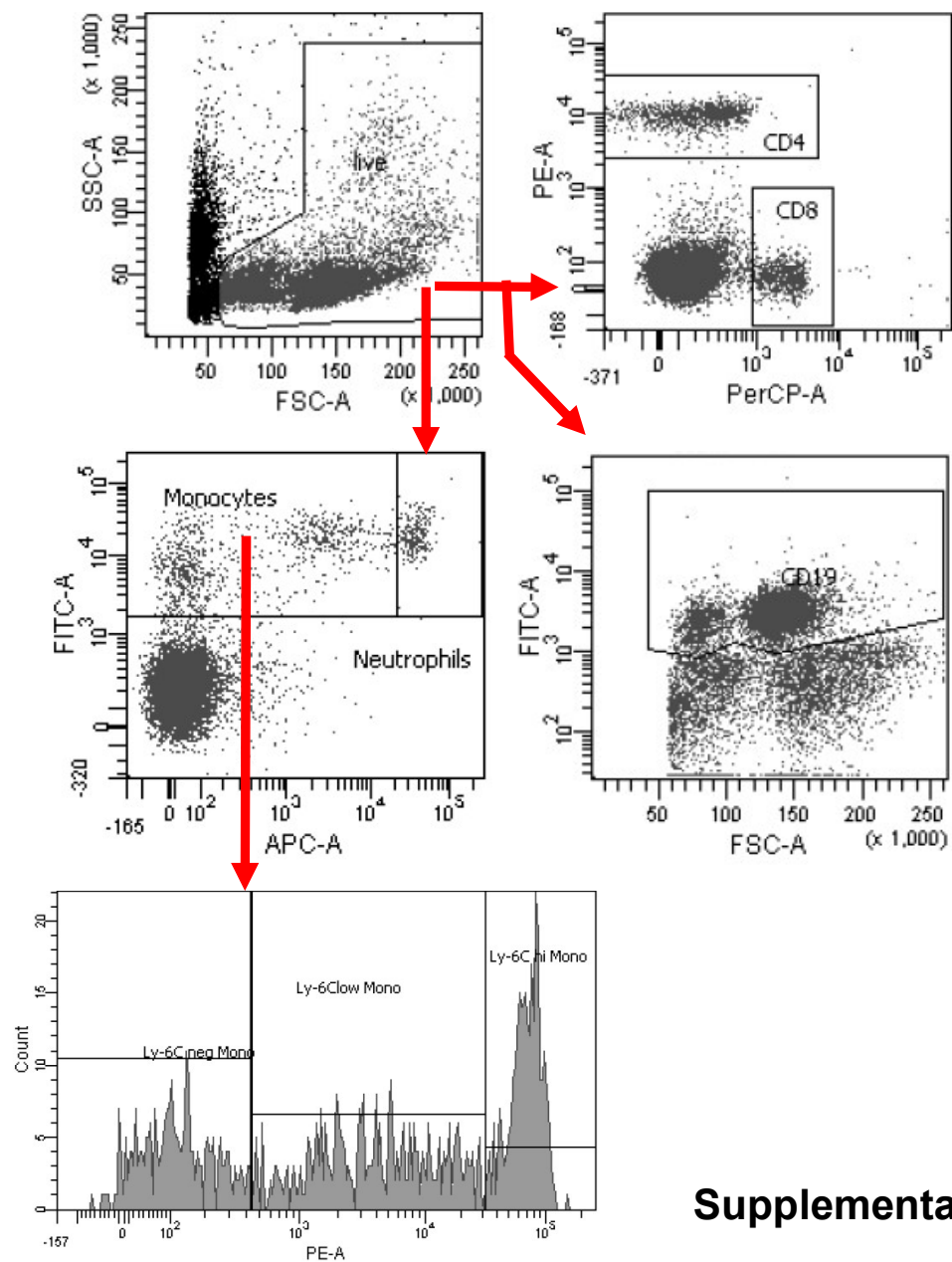

**Supplemental Figure 1: Gating strategy used for flow cytometric analysis**

Supplement: Supplementary file 1 — Supplementary Figure 1. [file 41598_2021_95858_MOESM1_ESM.pdf]
